# Comparative Performance Analysis of Commercial SARS-CoV-2 RNA Detection Assays: Implications for Sensitivity, Specificity, Accuracy, and Diagnostic Response Time

**DOI:** 10.3390/diagnostics16101554

**Published:** 2026-05-20

**Authors:** Adriana Guimarães dos Santos, José Rodrigo Santos Silva, Maria Luísa Rodrigues Nolasco, Marcus Vinicius de Aragão Batista

**Affiliations:** 1 Laboratory of Molecular Genetics and Biotechnology (GMBio), Department of Biology, Center for Biological and Health Sciences, Federal University of Sergipe, São Cristóvão 49107-230, SE, Brazil; drikaaguimaraes@hotmail.com (A.G.d.S.); luisanolasco1@gmail.com (M.L.R.N.); 2 Department of Statistics and Actuarial Sciences, Center for Exact Sciences and Technology, Federal University of Sergipe, São Cristóvão 49107-230, SE, Brazil; rodrigo.ufs@gmail.com

**Keywords:** SARS-CoV-2, RT-qPCR, pandemic, COVID-19, diagnosis, sensitivity

## Abstract

**Background/Objectives:** In 2020, the world found itself in the midst of the SARS-CoV-2 pandemic. The virus has spread globally, resulting in over 779 million cases worldwide. In response to this crisis, there arose a critical need for diagnostic techniques capable of meeting the overwhelming global demand, including RT-qPCR as the gold standard due to its high sensitivity and specificity. However, RT-qPCR has its limitations, including susceptibility to factors such as inadequate sample collection, variations in viral load, and insufficient clinical validation, all of which can lead to false negatives. Consequently, this study aims to evaluate the clinical performance of four commercial RT-qPCR kits for detecting SARS-CoV-2. **Methods:** The study utilized 200 nasopharyngeal swab samples collected in January 2022, comparing kits from Qiagen, Seegene, Bio-Manguinhos, and IBMP. **Results:** Results indicated significant differences in kit performance, with 66% of samples showing consistent results across all kits, and 34% showing discrepancies. Ct values were also analyzed, and statistical tests highlighted varying sensitivities among the kits, ranging from 100% to 86.82%. **Conclusions:** The study underscores how extraction and purification processes, kit quality, and target gene adequacy critically influence kit performance, influencing the occurrence of false positives and negatives.

## 1. Introduction

The pandemic caused by the novel coronavirus SARS-CoV-2 has had a profound global impact since its emergence in 2019, resulting in more than 779 million confirmed cases worldwide [1]. In Brazil alone, over 39 million cases and more than 716,000 deaths have been reported, according to official government data [2]. Due to its high transmissibility and rapid global spread, COVID-19 was declared a pandemic by the World Health Organization (WHO) in 2020 [3].

Following this declaration, several public health measures were implemented worldwide to reduce viral transmission [4], including social distancing, quarantine and frequent hygiene practices, as the main routes of transmission involve inhalation of aerosols or respiratory droplets and contact with contaminated surfaces [5]. In this context, larger respiratory particles (generally >5–10 μm) tend to settle rapidly due to gravity, thereby limiting transmission to short distances and direct contact, whereas smaller aerosols (≤5 μm) can remain suspended in the air for prolonged periods, disperse through airflow, and travel longer distances, being inhaled deeply into the respiratory tract. This distinction reflects different transmission dynamics, with one pathway predominantly associated with close-range exposure and direct droplet deposition, and another with a potentially airborne nature and broader reach related to aerosols [6]. In parallel, unprecedented efforts have been made by scientists and healthcare professionals worldwide to develop vaccines, therapeutic strategies and diagnostics tools capable of containing the spread of the virus and addressing the increasing demands on healthcare systems, with significant impacts on the global economy and society as a whole [3].

Patients affected by SARS-CoV-2 infection can present a variety of symptoms, but the virus has an affinity for the respiratory tract [7]. The most common symptoms include cough, headache, nausea, vomiting, fever and diarrhea. Although these manifestations are usually mild in most people, the disease can present greater severity in elderly patients and those with comorbidities, potentially leading to Severe Acute Respiratory Syndrome (SARS), which is associated with a relatively high mortality rate [7].

SARS-CoV-2 is an enveloped, single-stranded RNA virus belonging to the β-coronavirus genus within the *Coronaviridae* family, possessing one of the largest viral genomes known [8]. Its genome exhibits the typical organization of β-coronaviruses, comprising multiple open reading frames (ORFs), including ORF1a and ORF1b, which encode 16 nonstructural proteins (nsp1–nsp16) essential for viral RNA replication. Among these, the RNA-dependent RNA Polymerase (RdRP) corresponds to non-structural protein 12 (nsp12), encoded within the ORF1a/ORF1b region, and plays a central role in viral RNA replication and transcription, being highly conserved among coronaviruses. Furthermore, the viral genome encodes four main structural proteins, spike (S), nucleocapsid (N), envelope (E), and membrane (M), that play fundamental roles in viral entry, assembly, and packaging, as well as several accessory proteins that contribute to viral adaptation and interaction with the host [9].

These genomic features, particularly the high mutation rate characteristic of RNA viruses, contribute to the continuous emergence of new variants, directly influencing vaccination strategies and the development of diagnostic approaches. With the advancement of vaccination and the gradual return to normal activities, the genetic variability of SARS-CoV-2 has driven the development of increasingly effective vaccines against the new strains [7]. In parallel, diagnostic methods have evolved with the progression of the pandemic, initially using serological techniques to detect IgG and IgM, moving on to the widespread use of RT-qPCR, considered the gold standard for SARS-CoV-2 detection, given its greater sensitivity and specificity, and frequently complemented by antigen-based biosensors [10].

However, the rapid development of commercial RT-qPCR kits, often with limited clinical validation, has raised questions about the performance of these tests given the significant number of false negatives observed [11]. Moreover, the high frequency of mutations, especially in the spike (S) gene, may compromise the accuracy of assays targeting this region [12], further reinforcing the need for continuous evaluation of diagnostic tools.

Given that SARS-CoV-2 infection remains a global public health concern, and considering the ongoing emergence of viral mutations that may impact the sensibility and the specificity of molecular diagnostics, there is a critical need to assess the performance of available diagnostic methods. In this context, this study aims to comparatively evaluate different commercial RT-qPCR-based kits for SARS-CoV-2 diagnosis, in terms of sensitivity, specificity, accuracy and diagnostic response time, in nasopharyngeal swab samples obtained from Northeastern Brazil.

## 2. Materials and Methods

### 2.1. Ethical Considerations

All experiments were performed in accordance with the ethical standards of the Declaration of Helsinki. The study was approved by the Research Ethics Committee of the Federal University of Sergipe, under protocol number 5.529.359, approved on 15 July 2022. Patient consent was waived by the Institutional Research Ethics Committee due to the use of anonymized samples obtained from an existing hospital biobank, and because it was not feasible to contact the individuals who originally provided the samples.

### 2.2. Samples

In total, 200 nasopharyngeal swab samples immersed in 3 mL of 0.9% sodium chloride were collected at Hospital Primavera in the state of Sergipe, Northeastern Brazil, and stored at −70 °C for viral preservation. The use of 0.9% sodium chloride as a transport medium was adopted as an alternative to standard Viral Transport Medium (VTM) due to supply shortages during the COVID-19 pandemic. Immediately after collection, samples were stored at −20 °C; if analysis was not performed within 24 h, they were subsequently transferred to −70 °C for longer-term storage. Although the exact time interval between sample collection and initial storage could not be determined for each sample, all specimens followed this standardized handling protocol. The samples were previously used to perform RT-qPCR for SARS-CoV-2 in patients with suspected infection. Subsequently, the same samples were evaluated using different commercial RT-qPCR kits for SARS-CoV-2.

### 2.3. Description of the Amplification Kits for the Diagnosis of SARS-CoV-2

Four different kits were used to detect SARS-CoV-2, three of which include the RNA extraction step and two of which do not. Kit 1 is the Seegene Allplex™ SARS-CoV-2 Assay (Seegene, Votorantim, Brazil), which detects four virus-specific genes, the N, RdRP, E, and S genes. This kit can be used both with the RNA pre-extraction step and without the extraction step, with different protocols for each occasion. Kit 2 is the QIA Prep&Amp Viral RNA UM Kit (Qiagen, Hilden, Germany). It is a rapid detection kit, using just one reagent called Prep Buffer to prepare the reaction for the amplification step, spending just two minutes on this process and amplifying just one specific gene for SARS-CoV-2, RdRP. Kit 3 is the Molecular SARS-CoV-2 (EDx) kit (Bio-Manguinhos Institute, Rio de Janeiro, Brazil), which detects only one SARS-CoV-2-specific gene, the E gene. Kit 4 is the BIOMOL OneStep/COVID-19 (IBMP, Curitiba, Brazil), which detects two SARS-CoV-2-specific genes, the N, and ORF-1ab genes.

### 2.4. RNA Extraction

This study used Quick-DNA/RNA™ Viral MagBead extraction kit (Zymo Research, Orange, CA, USA), which uses magnetic beads to separate and purify DNA or RNA from a sample, according to the manufacturer information.

### 2.5. Experimental Design

The 200 nasopharyngeal swab samples were selected according to the results already known using the QIA Prep&Amp Viral RNA UM Kit (Qiagen, Hilden, Germany). It is important to note that the entire process, both extraction and amplification, was carried out by a single operator. The samples were identified only by barcode, without identifying any patient personal data. In total, 117 samples detectable for SARS-CoV-2 and 83 samples not detectable for SARS-CoV-2 were used, following the interpretations provided by the Qiagen kit. The samples were divided into eight groups, with the eighth and final group being for repeats only, i.e., samples that did not obtain a result due to some failure in the RT-qPCR reaction or in the extraction stage.

Each group contained 31 samples to be extracted, and took an average of 1 h and 40 min for each cycle of 8 samples. Only 8 samples were extracted each cycle to avoid contamination as the eppendorfs are very close to each other. RT-qPCR reactions were performed using a QuantStudio 5 Real-Time PCR System (Thermo Fisher Scientific, Waltham, MA, USA) equipped with a high-intensity white LED module (OptiFlex™, Thermo Fisher Scientific, Waltham, MA, USA). A summary of the technical specifications of each assay is provided in Supplementary Table S1. A negative control was added to each extraction group of 31 samples, RNase-free water (Thermo Fisher Scientific, Waltham, MA, USA, catalog number FERA57775) was used instead of a sample as a reaction control, ensuring that there was no contamination. In groups 1 and 2, one qPCR was carried out for each kit with 62 samples, totaling three qPCRs for each 62 samples. In groups 3 and 4, one qPCR was carried out for each kit with 62 samples, totaling three qPCRs for each 62 samples. In groups 5, 6, and 7, only one qPCR of each kit was carried out for 76 samples. In the last group (group 8), 6 samples had their PCR test repeated. Table 1 details the experimental design described.

To divide the groups, the Seegene protocol without extraction was divided into just 3 groups, since it does not require prior extraction and is a faster protocol. The amplification plate holds 94 samples + 1 negative control and 1 positive control, so groups 1 and 2 had 94 samples, and group 3 had 12 samples + 8 repeats of the two previous PCRs (Table 2).

### 2.6. Specificity of Each RNA Amplification Kit

According to the manufacturers protocols, the Seegene Allplex™ SARS-CoV-2 Assay kit (Seegene, Votorantim, Brazil) stands out for detecting 4 targets at the same time, in addition to the presence of an Internal Control (IC) which guarantees greater safety for the reaction. The genes detected are gene E detected by the FAM fluorochrome, gene RdRP and gene S detected by the same ROX fluorochrome, and gene N detected by the Cy5 fluorochrome, in addition to the Internal Control detected by the VIC. The reaction takes approximately 122 min. For the run to be valid, the Ct of both the Negative Control and the samples must be less than or equal to 40.

The SARS-CoV-2 EDx kit (Bio-Manguinhos Institute, Rio de Janeiro, Brazil) detects only one target, the E gene and an internal control called RP. The E gene is detected by the FAM fluorochrome, and the RP control is detected by the VIC fluorochrome. The reaction lasts approximately 77 min. For the run to be valid, the Ct of the Negative Control must be less than or equal to 40, and of the positive control less than or equal to 37. Samples must have a Ct ≤ 40 for the E gene, and a Ct ≤ 35 for the RP internal control.

The SARS-CoV-2 IBMP kit (IBMP, Curitiba, Brazil) detects two targets, the N gene and the ORF 1ab gene, as well as an Internal Control (IC). The N gene is detected by the VIC fluorochrome, the ORF 1ab gene is detected by the FAM fluorochrome, and the Internal Control is detected by the ROX fluorochrome. The reaction takes approximately 131 min. The Ct of the Positive Control must be less than or equal to 35 for the run to be valid, while the Ct of the samples must be up to 40.

Qiagen’s QIA Prep&Amp Viral RNA UM Kit (Qiagen, Hilden, Germany) is extraction-free and only detects one target, the RdRP gene, detected by the FAM fluorochrome, and has the presence of two internal controls, RNA IC detected by the Cy5 fluorochrome, and HS IC detected by the VIC fluorochrome. The reaction lasts approximately 55 min. The Ct limit for Human Sample Control (HS IC) must be less than or equal to 35 and Internal Control (RNA IC) covers a range between 30 and 34 for the run to be valid, while the Ct value of the samples is not indicated by the manufacturer, so in this study it was considered valid up to 40.

### 2.7. Statistical Analysis

Exploratory data analysis was carried out by calculating simple and percentage frequencies. The bivariate inferential analysis used McNemar’s test [13] to associate the results considering the positive tests from at least 3 different kits. Logistic regression models [14], with a log link function, were used to check the quality of the gold standard’s (RT-qPCR) predictability, from the results of different commercial RT-qPCR kits for SARS-CoV-2, in which the statistics of sensitivity, specificity, accuracy, positive predictive value, negative predictive value and the area under the ROC curve (AUC) were obtained [15,16,17,18]. The Ct values for the cases with a detectable result were compared using the Kruskal–Wallis test [19], with Dunn’s post hoc [20] (with Bonferroni’s correction), and the results were represented graphically using the Boxplot chart.

The data was organized in the Microsoft Excel (Microsoft 365, Office) program, and all statistical analyses were carried out in the R software, version 4.3.1 [21]. The significance level adopted throughout the study is 5%.

## 3. Results

In total, 200 nasopharyngeal swab samples were analyzed. All the samples were subjected to the same extraction process. The Seegene Allplex™ kit was also tested with the protocol without extraction. The necessary repetitions were checked, and the samples that had their reaction invalidated during any of the runs were repeated in their respective kits. All the reactions were carried out with at least one negative control and one positive control to ensure that the reactions were not contaminated. The samples were chosen at random according to the previous results using the QIA Prep&Amp kit (Qiagen, Hilden, Germany), in which they were classified in either detected or not detected for SARS-CoV-2. In this context, 117 detectable samples (58.5%) and 83 non-detectable samples (41.5%) were used.

**Figure 1:**
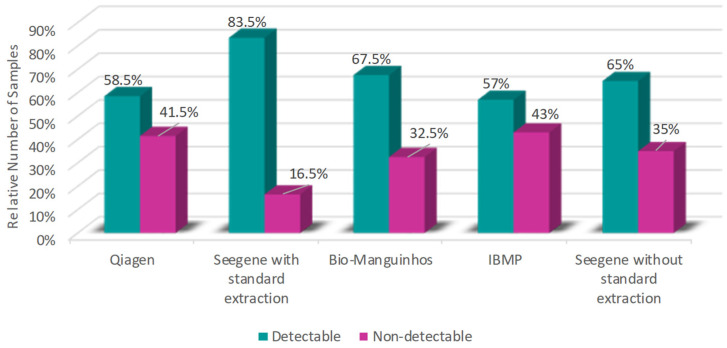
Percentage of detectable and non-detectable samples from each kit. In blue, the percentage of positive samples, and in orange the percentage of negative samples, considering the total number of 200 samples.

Figure 1 shows the number of detectable and non-detectable samples for SARS-CoV-2 from each kit. The highest number of positive samples is from the Seegene kit (Seegene, Votorantim, Brazil) with standard extraction, with 164 (83.5%) detectable and 36 (16.5%) non-detectable samples. This was followed by the Bio-Manguinhos kit (Bio-Manguinhos Institute, Rio de Janeiro, Brazil), which showed 135 (67.5%) detectable and 65 (32.5%) non-detectable samples. The Seegene kit (Seegene, Votorantim, Brazil) without standard extraction presented 130 (65%) detectable and 70 (35%) non-detectable samples. With a lower number of detectable samples is the Qiagen kit (Qiagen, Hilden, Germany), which does not have a specific extraction protocol, with 117 (58.5%) detectable and 83 (41.5%) non-detectable samples. The IBMP kit (IBMP, Curitiba, Brazil), whose protocol involves standard extraction, had 114 (57%) detectable and 86 (43%) non-detectable samples.

**Figure 2:**
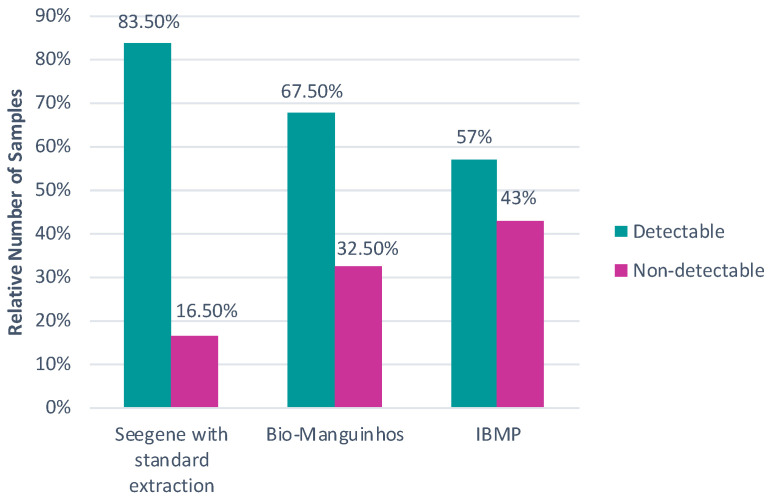
Percentage of detectable and non-detectable samples from kits that use the standard extraction protocol (Seegene Allplex™, Bio-Manguinhos, and IBMP kits), showing higher positivity in the Seegene kit and lower positivity in the IBMP kit.

Only the kits with the standard extraction protocol were compared in Figure 2, with a considerable number of differences among them, despite the fact that all three were carried out using the same protocol and a single extraction. While the Seegene kit (Seegene, Votorantim, Brazil) presented 83.5% positive samples, Bio-Manguinhos (Bio-Manguinhos Institute, Rio de Janeiro, Brazil) had around 67.5% positive samples, and IBMP had only 57% positive samples.

**Figure 3:**
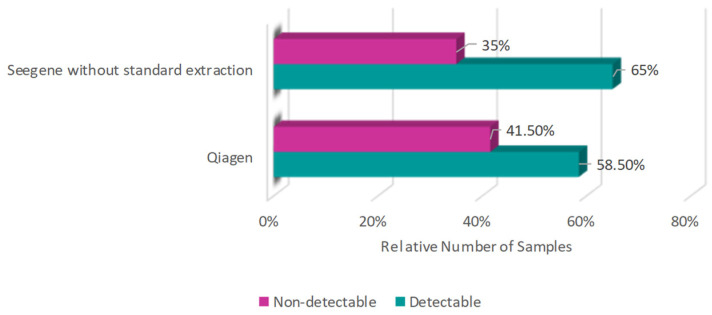
Percentage of detectable and non-detectable samples using kits without the standard extraction step (Seegene Allplex™ and Qiagen), with numbers relatively close to each other.

In a comparison between the kits that do not have a standard extraction protocol, 65% samples were positive in the Seegene kit (Seegene, Votorantim, Brazil) without standard extraction, while using the Qiagen kit (Qiagen, Hilden, Germany) there were 58.5% detectable samples (Figure 3).

**Figure 4:**
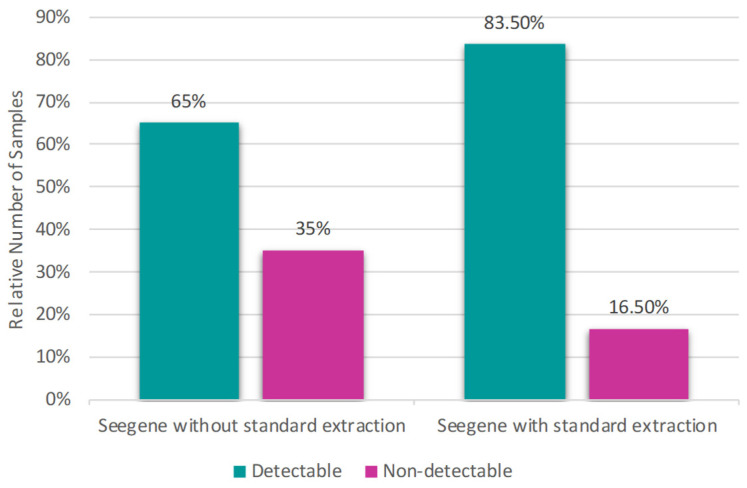
Percentage of detectable and non-detectable results using the Seegene Allplex™ kit (Seegene, Votorantim, Brazil) with and without the standard extraction step, with a higher positivity rate (83.50%) in the protocol with the standard extraction, while the protocol without standard extraction showed a positivity rate of 65%.

The comparison between the Seegene kit (Seegene, Votorantim, Brazil) using the protocols with standard extraction and without standard extraction showed more significant differences: while Seegene (Seegene, Votorantim, Brazil) with standard extraction had 83.50% detectable and 16.50% non-detectable samples, the Seegene kit (Seegene, Votorantim, Brazil) without standard extraction presented 65% detectable and 35% non-detectable samples (Figure 4).

When comparing the number of repeats of each kit, it is possible to observe that kits that use standard extraction have a lower number of repeats than kits that do not use the standard extraction, as the latter is more subject to various interferences since it does not go through a complete RNA purification process. Repeats are determined according to each manufacturer’s table of result interpretations, and are necessary when the Internal Control of the specific reaction does not amplify. The whole process is then carried out again, extraction (if the kit has this step) and amplification. In this context, the Seegene kit (Seegene, Votorantim, Brazil) with the standard extraction protocol required 5 (2.5%) repetitions, while the Bio-Manguinhos kit (Bio-Manguinhos Institute, Rio de Janeiro, Brazil) only required 3 (1.5%) repetitions. The IBMP kit (IBMP, Curitiba, Brazil) required 4 (2%) repetitions and the Seegene kit (Seegene, Votorantim, Brazil) without standard extraction required 9 (4.5%) repetitions.

**Figure 5:**
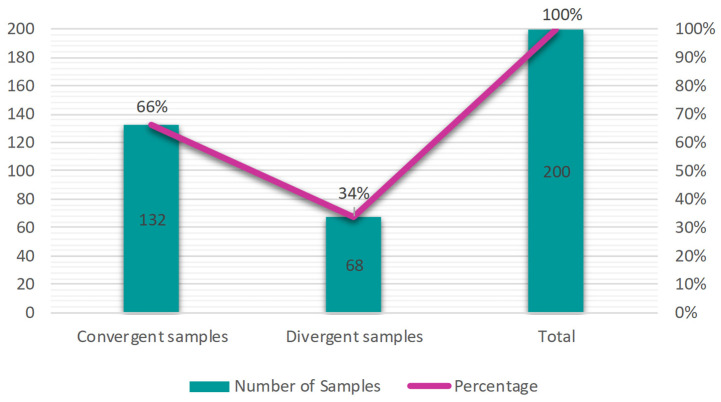
Percentage of convergent and divergent samples among all the kits. Samples that obtained the same result in all the kits were considered convergent, if not they were considered divergent. Convergence was obtained for 66% of samples, and divergence for 34% of samples.

At the end of the comparison among all kits, 66% of samples presented convergent results and 34% of samples with divergent results, considering convergent when the result is the same in all the kits and divergent when there is at least one different result between all the kits (Figure 5).

Table 3 shows the comparison between the kits with the gold standard (those that had the same result in at least 3 out of the 5 assays), taking into account the detected and undetected samples. The Qiagen kit presented 112 (97.4%) detected results that agreed with at least three different kits, and 68 (80%) undetected results that agreed with at least three kits. In contrast, the Qiagen kit presented divergent results for 17 (20%) tests that were detected in other kits, and three (2.6%) tests were not detected in other kits but detected in the Qiagen kit (*p* = 0.004). The differences were also significant in the Qiagen (*p* = 0.004), the Seegene with standard extraction (*p* < 0.001) and the IBMP (*p* = 0.001) kits, but not in the Bio-Manguinhos (*p* = 0.181) and in the Seegene without extraction (*p* = 1.000) kits.

**Figure 6:**
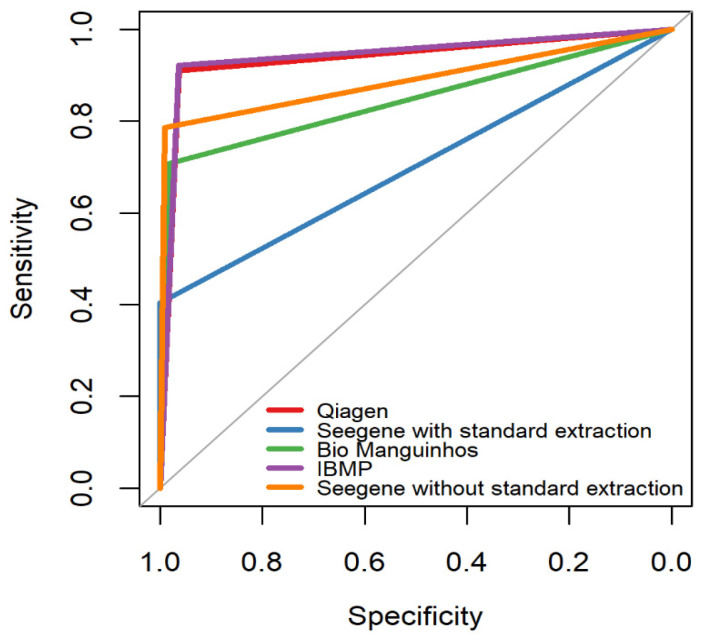
ROC curve between sensitivity and specificity in the comparison between all the kits evaluated in this study.

Table 4 also shows the percentages of sensitivity, specificity, accuracy, positive predictive value, negative predictive value and the area under the ROC curve (AUC) using logistic regression. It is noticeable that the Seegene kits with extraction and the Bio-Manguinhos kit (Bio-Manguinhos Institute, Rio de Janeiro, Brazil) perform better in terms of sensitivity, with 100% and 96.90% respectively, but there is a big drop, especially in the Seegene kit with extraction, in relation to specificity (50.70%) compared to kits such as Qiagen (95.77%) and IBMP (97.18%). The accuracy values, the weighting between the sensitivity and specificity values, are balanced between all the kits, unlike the positive predictive value and negative predictive value. The AUC, or area under the ROC curve, is shown in both Table 4 and Figure 6.

Table 5 shows the statistics for the Cts values of the RdRP, E, N, RdRP/S and ORF-1ab targets for each kit separately, calculating the number of samples in which that target was detected, the median Cts, the standard deviation, the coefficient of variation and the minimum and maximum number detected for each target. It is noticeable that the median of the N, RdRP/S targets in the Seegene kit in both methodologies is higher, as is the E gene in the Bio-Manguinhos kit. The highest number of Ct (maximum Ct) were also found in these same targets.

The Cohen’s Kappa coefficient of agreement, which shows how much the results of each kit agreed, was assessed (Table 6). Each Kappa value indicates a level of agreement and also a percentage. From 0 to 0.20 the level of agreement is considered none (from 0 to 4%), from 0.21 to 0.39 the level of agreement is minimal (between 4 and 15%), from 0.40 to 0.59 the level of agreement is considered weak (from 15% to 35%), from 0, 60 to 0.79 the level of agreement is moderate (from 35 to 63%), from 0.80 to 0.90 the level of agreement is strong (between 64% and 81%) and finally numbers above 0.90 have an almost perfect level of agreement (from 82% to 100%). It can therefore be seen that the levels of agreement were classified from moderate to strong, with the highest levels between the Qiagen and IBMP kits (0.77), and the Qiagen and Seegene without extraction kits (0.71).

## 4. Discussion

The RT-qPCR technique is considered the gold standard for SARS-CoV-2 detection, being used both as one of the criteria for diagnosis definition and for hospital discharge, while also assisting in determining appropriate treatment [11]. However, despite all its advantages, the technique also presents limitations and is primarily subject to false negatives, given that its sensitivity can be affected by multiple factors, such as days of symptom onset, collection method, viral load, sample handling, and the performance of the kits used [22]. Different kits were approved for emergency use during the pandemic, but in non-emergency situations, this type of kit would require 3 to 5 years of study to verify efficacy across a large number of clinical samples with ongoing optimizations, thereby compromising assay performance [11].

All the kits used in this study were approved by the competent regulatory authorities for emergency use during the pandemic and are all still in use, including the two kits manufactured in Brazil (Bio-Manguinhos and IBMP).

The primary objective of this study was to conduct a comparative analysis of four RT-qPCR kits for SARS-CoV-2 diagnosis using randomly selected clinical samples with a broad range of Cts values, so that the comparison would be as realistic as possible within a laboratory routine, encompassing patients with different viral loads seeking accurate diagnosis. Selecting the most appropriate kit for each routine is of great importance, taking into account cost-effectiveness, diagnostic turnaround time, and result accuracy. Significant variability was observed among the kits across the 200 samples analyzed, especially in cases with low viral load.

In the comparative analysis by Gdoura et al. (2022) [23], which compared four different kits using 260 nasopharyngeal swab samples, 70.8% (184) of the samples were concordant and 29.2% (76) were discordant, considering samples that detected all targets with Ct values up to 39. Among the discordant samples, the Ct values of positive targets ranged from 13 to 39, with a mean of approximately ±33, with the N gene considered the most problematic due to its high susceptibility to mutations, especially following the emergence of the Alpha variant. The study also associates high Ct values in the N gene with likely false-positive results or late positivity signals in convalescent patients. In this study, concordance and discordance values were very similar, with 66% (132) of samples being concordant and 34% (68) discordant, considering Ct values of positive samples up to 40, ranging from 12.42 to 39.18. The coefficient of variation in the N gene differed across each kit but also exhibited higher median values compared to the other targets, ranging from 28.60 in the Seegene kit with extraction to 28.85 in the Seegene kit without extraction. An exception to these values is the median of the IBMP kit (26.59), given its lower sample positivity rate (114), compared to N gene positivity in the Seegene kit with extraction (154) and without extraction (125).

In another study by Rangaiah et al. (2021) [24], the concordance profile was 81.33% for detected samples and 18.67% for undetected samples, based on a comparison of 12 different commercial kits using 75 samples, with target Ct values differing significantly among the 12 kits. One of the kits used was Seegene’s Allplex 2019-nCoV, the previous version of the Seegene Allplex™ SARS-CoV-2 Assay, which obtained a sensitivity of 98.36% and specificity of 100%. Significant differences were found between the Ct values of the E, N and ORF-1ab genes (*p* < 0.001), whereas no such significance was observed between the S and RdRP genes. Prior to interpreting assay-specific performance differences, it is important to consider the reference standard adopted in this study. Since the primary objective was a comparative evaluation of different commercial RT-qPCR kits for SARS-CoV-2 detection, a consensus-based reference standard, defined as concordance among at least three assays, was used as an operational comparator in the absence of an independent confirmatory method, such as viral genome sequencing or clinical diagnosis. Although this strategy has been widely employed in comparative diagnostic studies [25,26] it is not without limitations. In particular, it introduces elements of circular reasoning, as the assays under evaluation contribute to the definition of the reference standard and may favor concordant results, potentially leading to overestimation of sensitivity and specificity [26,27,28]. Furthermore, assays with higher analytical sensitivity may be disadvantaged, as samples detected by a single method may be classified as false positives despite representing true infections with low viral loads [27].

In this study, both Seegene Allplex™ methodologies, with and without standard extraction, were employed. The sensitivity and specificity of the kit with standard extraction were 100% and 50.70% respectively, while the values observed for the extraction-free methodology were 93.02% sensitivity and 87.32% specificity. Compared to the other kits, the difference in the Seegene with standard extraction was statistically significant (*p* < 0.001), whereas the Seegene without standard extraction was not (*p* = 1.000). The relatively low specificity associated with the extraction-based approach requires careful interpretation. One possible explanation is the occurrence of false-positive results due to non-specific amplification, cross-reactivity, or laboratory contamination. However, these factors could not be directly assessed in the absence of a viral interference panel. Alternatively, this finding may reflect the higher analytical sensitivity of the assay compared to the consensus-based reference standard used in this study. In this context, samples detected exclusively by the Seegene assay may represent true positives with low viral loads that were not identified by the other kits, rather than false-positive results. Notably, the use of a consensus-based reference standard may disproportionately penalize more sensitive assays, as discordant results are systematically classified as false positives. Therefore, the reduced specificity observed may reflect methodological limitations rather than an intrinsic limitation of the assay itself [27,28].

Fukasawa et al. (2021) [29] evaluated 205 nasopharyngeal and oropharyngeal swab samples compared using seven SARS-CoV-2-specific RT-qPCR assays, including the Seegene Allplex™ SARS-CoV-2 Assay, the Bio-Manguinhos SARS-CoV-2 (EDx) and the BIOMOL OneStep/COVID-19 IBMP. The mean sensitivity values ranged from 83.6% and 100%, with the Seegene Allplex™ kit having a sensitivity of 97.3%, the Bio-Manguinhos kit 87.7%, and the IBMP kit 87.7%. In the present study, the sensitivity of these assays was 100%, 96.9% and 86.8% respectively, noting that Fukasawa et al. (2021) [29] used the Seegene Allplex™ kit with standard extraction. No significant differences were found in relation to the specificity of the kits.

Fukasawa et al. (2021) [29] also describe that mean Ct values range from 27 to 31, with the lowest coefficient of variation found for the RdRP gene, identified as the least sensitive target. In this study, mean Ct values ranged from 23 to 30, with the lowest coefficient of variation found for the RdRP/S target (15.02), and the highest coefficient of variation found for the N gene (24.94). Fukasawa et al. (2021) [29] showed that the median was similar for all the assays, only significant divergences were found between the RdRP target of two kits tested, the in-house IAL assay and Seegene and between the E gene of Bio-Manguinhos and Seegene. This is similar to our results that also presented a significant difference between the mean RdRP targets of the Qiagen kit and the Seegene kit with and without standard extraction and between the E gene of Bio-Manguinhos and Seegene with and without standard extraction, with the addition of the N gene which also showed differences between the Seegene kits with and without standard extraction and the IBMP kit. The two Seegene methodologies showed very similar Ct values.

The WHO [1] considered that results detected for SARS-CoV-2 should be considered with the presence of at least two specific target genes or with the presence of one target gene detected with a subsequent sequencing of the virus; however, this decision was revised shortly afterwards by PAHO that single target tests could be used with the presence of the E gene [29]. The Bio-Manguinhos kit, which only has the E gene as a detectable target, had 96.90% sensitivity in this study, considering that the variant circulating at the time the samples were taken, in July 2022, was probably the Omicron BA.2 lineage, which was the circulating variant most present in Brazil at that time, according to data from the Oswaldo Cruz Foundation’s Genomic Network [30]. This variant has mutations mainly in amino acids of the S protein, with no evidence of significant mutations in the N protein [31], which explains the high sensitivity of this kit. Fukasawa et al. (2021) [29] also mention that there is great genomic diversity in the RdRP and N genes among the various existing variants, unlike the E gene. However, the kit does not have a safety margin in case other variants with mutations in the E gene appear, considering that SARS-CoV-2 is a virus with high mutation rates [31].

Gdoura et al. (2022) [23] also reported that the E gene in the protocol used for the BERLIN kit was a target gene that remained conserved and proved to be more sensitive compared to the RdRP gene, which had a median of 28.9, while the RdRP gene had a median of 30.5. This was also the case in our study, since the median of the RdRP gene ranged from 29.70 to 29.78, while the median of the E gene ranged from 26.70 to 26.43 in the Seegene kit, showing that in this kit the E gene was more sensitive than the RdRP gene. What differs in our study are the medians of the RdRP gene in the Qiagen kit (23.06), which are considerably lower compared to all the other kits. This can be explained by the low sensitivity of the kit, considering that the maximum Ct amplified by it was 34.94.

Shen et al. (2020) [32] compared 94 positive samples for SARS-CoV-2 in six different commercial kits, excluding samples with a Ct value greater than 35. Their sensitivity ranged from 100% to 90.4%, with the positive predictive value ranging from 100% to 95.74% and the negative predictive value ranging from 100% to 87.67. In our study, the positive predictive value ranged from 98.25 to 78.66 and the negative predictive value ranged from 100% to 80%. The same study found that the RdRP gene is less sensitive than the ORF-1ab gene, and the values obtained in our study also show that the RdRP target gene is less sensitive than the ORF-1ab gene, as the kit can only detect Cts values of up to 35, while the ORF-1ab gene can detect Cts of up to 39.

Gdoura et al. (2022) [23] observed that Ct values greater than 28 can generate non-specific sequences due to inactivation of the Taq polymerase and suggested that values above 28 should be disregarded, but in our study the Ct value was considered following the guidelines of each supplier, in which samples with Ct up to 40 were considered detected.

Barros et al. (2022) [33] compared the Seegene Allplex™ SARS-CoV-2 Assay kit with the non-extraction methodology with a commercial kit with the present extraction methodology, suggesting that Cts values between 35 and 40 would be suggestive of false positives, recommending that only Ct values up to 35 should be considered. In our study it was observed that this specific kit has higher Cts values compared to the other kits, but if Cts values above 35 were not considered, false negative results would be generated, since even with high Cts values the results in the other kits used for comparison were positive.

Barros et al. (2022) [33] also compared results with maximum Ct values between 35 and 40. When the Ct value was increased to 40, samples that were not considered positive became so, mainly due to the N gene, which showed maximum Ct values of up to 39.89. The same happened in our study, with the maximum Ct of the N gene being 39.40. If we compare the number of samples detected for the N gene in the Seegene kit with extraction, we get 154 samples, while in Seegene without extraction we observe 125 samples, which corroborates the low specificity of Seegene with extraction, indicating that the percentage of samples detected in this kit may have been generated by false-positive results.

Barros et al. (2022) [33] also demonstrated a correlation between the target genes, observed between genes E and RdRP, genes E and N, and genes N and RdRP. In our study, the kits with the highest correlation are the IBMP and Qiagen, and Seegene without extraction and Qiagen, which have moderate/strong levels of agreement. It is possible to observe which kits have the best balance between sensitivity and specificity, led by the Qiagen and IBMP kits, followed by Seegene without extraction and Bio-Manguinhos, and ending with the Seegene kit with extraction.

The repetitions carried out were of reactions that did not amplify the internal control, the samples that may have been considered inconclusive by the respective kit were considered negative or positive depending on the analysis of the curve, the Ct and what each kit determines in its interpretation, and there were no inconclusive results or repetitions for these results. In this way, it was possible to determine the number of repetitions that each kit required, observing a greater number in the Seegene kit without extraction (9) compared to the methodologies with standard extraction. According to Fenaux et al. (2022) [34], these repetitions are expected in kits without extraction because the RNA does not undergo the purification process, so the reaction suffers from many more interferents that can inactivate the PCR.

Fenaux et al. (2022) [34] determined the performance of the QIA Prep&Amp Viral RNA UM Kit, a method without extraction that has a great advantage due to its short diagnostic time. In this analysis, 156 nasopharyngeal swab samples in different transport media were submitted for evaluation, and 8 out of the 156 (around 5.1%) samples did not amplify the internal control, making it necessary to repeat the reaction. The sensitivity of the results was 100% for samples with Ct < 34, 56.7% for Ct between 34 and 38 and 11.1% for Ct > 38. In our study, the QIA Prep&Amp kit also proved to be less sensitive in samples with higher Cts, with a coefficient of variation of 24.94, the highest detectable Ct being 34.94, below the Cts of genes in the Seegene kit where the RdRP target with extraction detected a maximum Ct of 42.37 and Seegene without extraction detected a maximum Ct of 41.50. The study concludes that this method can be used as a backup in laboratories, replacing safer protocols should the need arise.

Beyond the observed analytical performance, it is important to emphasize that RT-qPCR assays detect viral RNA, which does not necessarily confirm the presence of a viable or infectious virus. Consequently, positive results may represent residual or non-infectious viral genetic material, particularly in individuals in advanced stages of infection or after viral clearance. Moreover, non-specific amplification and environmental contamination remain potential sources of false-positive signals, especially at elevated Ct values. In this study, positivity was defined based on Ct values according to the manufacturer recommendations, without independent clinical confirmation. The absence of longitudinal clinical data, including symptomatology or serological evidence, limits the ability to distinguish true infection from potential false-positive outcomes [35,36]. Accordingly, the findings should be interpreted as an analytical comparison of assay performance, rather than a conclusive assessment of clinical diagnostic accuracy.

Although the results of this study are highly relevant for evaluating the performance of the kits in a clinical samples cohort, some limitations must be acknowledged. The absence of serial dilution experiments limited the ability to quantify extracted genetic material and to establish the analytical limit of detection (LOD) for each assay, which may impact the interpretation of sensitivity estimates. Additionally, no viral interference panels or cross-reactivity assays with other common respiratory pathogens were performed, limiting the evaluation of specificity-related factors. The selection of samples based on prior results obtained with a single assay introduces a potential selection bias, as samples detectable by other kits may have been excluded, potentially influencing sensitivity and specificity estimates. Furthermore, the lack of clinical follow-up data, including symptom progression and serological confirmation, were also unavailable, restricting the ability to assess diagnostic accuracy and distinguish true infection from potential false-positive results. Nevertheless, previous studies that incorporated analytical validation approaches have reported comparable performance patterns among RT-qPCR assays [27,28]. However, these studies were conducted under controlled laboratory conditions, whereas the present study reflects performance in real-world clinical samples. Therefore, our findings complement existing analytical data by providing evidence of assay behavior under routine diagnostic conditions.

## 5. Conclusions

The results of this study suggest that the quality of the kits is not linked to just one factor. The extraction step is an important factor for eliminating possible interferences from the reactions; however, the number of targets must also be taken into consideration, taking into account that kits that have a greater number of targets tend to be more sensitive than kits that only have one or two targets and considering that we have a large number of variants and possible mutations in any of the genes analyzed. In general, there are advantages and disadvantages to each kit, it is extremely important to regularly evaluate the protocols and kits used, adapting them to the current routine and considering the emergence of new variants. However, all kits have satisfactory sensitivity for detecting SARS-CoV-2 by RT-qPCR.

## Figures and Tables

**Table 1:** Division of groups for RNA extraction from nasopharyngeal swab samples.

Group of Samples	Number of Samples per Extraction	Number of Samples for PCR	Number of PCRs
1	31	62	3
2	31		
3	31	62	3
4	31		
5	31	76	3
6	31		
7	14		
8	6 (repetitions)	6	3

**Table 2:** Division of groups to perform the RT-qPCR using the Seegene Allplex™ SARS-CoV-2 Assay kit with the protocol without extraction.

Group	Number of Samples	Number of PCRs
1	94	1
2	94	1
3	12 + 8 repetitions	1

**Table 3:** Values and percentages of agreement and disagreement between the kits, taking into account detections and non-detections in at least 3 out of the 5 kits tested.

Variable/Category	Gold Standard		*p*-Value
	Detected	Not Detected	
Qiagen			
Detected	112 (97.4)	3 (2.6)	0.004
Not detected	17 (20.0)	68 (80.0)	
Seegene with extraction			
Detected	129 (78.7)	35 (21.3)	< 0.001
Not detected	0 (0.0)	36 (100.0)	
Bio-Manguinhos			
Detected	125 (92.6)	10 (7.4)	0.181
Not detected	4 (6.2)	61 (93.8)	
IBMP			
Detected	112 (98.2)	2 (1.8)	0.001
Not detected	17 (19.8)	69 (80.2)	
Seegene without extraction			
Detected	120 (93.0)	9 (7.0)	1.000
Not detected	9 (12.7)	62 (87.3)	

**Table 4:** Statistics of sensitivity, specificity, accuracy, positive predictive value, negative predictive value and the area under the ROC curve (AUC) using the logistic regression model of the McNemar test. The corresponding values are presented as percentages.

Statistics	Qiagen	Seegene with Extraction	Bio-Manguinhos	IBMP	Seegene Without Extraction
Sensitivity	86.82	100.00	96.90	86.82	93.02
Specificity	95.77	50.70	85.92	97.18	87.32
Accuracy	90.00	82.50	93.00	90.50	91.00
Positive predictive value	97.39	78.66	92.59	98.25	93.02
Negative predictive value	80.00	100.00	93.85	80.23	87.32
AUC	75.0	75.0	91.0	92.0	90.0

**Table 5:** The variation in Cts per target gene in each kit, including the median, standard deviation, coefficient of variation, minimum and maximum Ct values. Different letters mean statistical significative difference.

Assays and Their Targets		N	Mean	Standard Deviation	Coefficient of Variation	Minimum Ct	Maximum Ct
Qiagen	RdRP	112	23.06	5.47 d	23.74	13.58	34.94
Seegene with extraction	E	149	26.70	6.66 ab	24.94	14.66	39.49
	N	154	28.60	6.13 ac	21.44	16.21	39.18
	RdRP/S	158	29.70	5.95 c	20.02	17.50	39.77
Bio-Manguinhos	E	131	28.86	5.73 ac	19.85	18.60	39.93
IBMP	N	114	26.59	4.65 ab	17.50	16.57	36.97
	ORF-lab	112	26.23	5.02 b	19.13	16.86	38.99
Seegene without extraction	E	122	26.43	6.38 ab	24.12	12.42	38.28
	N	125	28.85	5.19 ac	17.98	20.22	39.40
	RdRP/S	120	29.78	4.47 c	15.02	21.76	39.58

**Table 6:** Cohen’s Kappa concordance coefficient, showing the concordance of the kit results with each other, highlighting the strong concordance between the Qiagen and IBMP kits, and the Qiagen and Seegene without extraction kits.

	Seegene with Extraction	Bio-Manguinhos	IBMP	Seegene Without Extraction
Qiagen	0.41 (0.30–0.53)	0.66 (0.56–0.77)	0.77 (0.67–0.86)	0.71 (0.61–0.81)
Seegene with extraction		0.55 (0.42–0.67)	0.45 (0.34–0.56)	0.50 (0.37–0.62)
Bio-Manguinhos			0.70 (0.60–0.80)	0.64 (0.53–0.76)
IBMP				0.68 (0.57–0.78)

## Data Availability

The data that support the findings of this study are available from the corresponding author upon reasonable request.

## Supplementary Materials

The following supporting information can be downloaded at: https://www.mdpi.com/article/10.3390/diagnostics16101554/s1, Supplementary Table S1: Technical specifications of the RT-qPCR assays evaluated for SARS-CoV-2 detection, including target genes, reaction volumes, and thermal cycling conditions.
