# Supplementary material for: Comparative Performance Analysis of Commercial SARS-CoV-2 RNA Detection Assays: Implications for Sensitivity, Specificity, Accuracy, and Diagnostic Response Time
Source: Diagnostics (Basel). 2026 May 20;16(10):1554. doi: 10.3390/diagnostics16101554 (PMC13205149; doi:10.3390/diagnostics16101554)
Supplement: Supplementary file 1 [file diagnostics-16-01554-s001.zip › diagnostics-4293866-supplementary.pdf]

Supplementary Table S1: Technical specifications of the RT-qPCR assays evaluated for SARS-CoV-2 detection, including target genes, reaction volumes, and thermal cycling conditions.

Parameter	Seegene Allplex	Seegene Allplex	QIA Prep&Amp Viral RNA UM	SARS-CoV-2 (EDx) BioManguinhos	BIOMOL OneStep / COVID-19 IBMP
**Target gene (s)**	gene N, RdRP, E and S	gene N, RdRP, E and S	Gene RdRP	Gene E	Gene N and ORF-1ab
**Sample volume**	20µl	20µl	10 µl	5 µl	5 µl
**Reagents volume**	SARS MOM: 5 µl<br>EM8: 5 µl<br>Water: 5µl<br>Total:15 µl	SARS MOM: 5 µl<br>EM8: 5 µl<br>Water: 4µl<br>RP-VIC 2: 1 µl<br>Total:15 µl	RNA Viral: 5 µl<br>Primer/Probe: 1 µl<br>IC RNA: 2 µl<br>IC HS: 1 µl<br>Water: 2 µl<br>Total: 10 µl	PCR Mix: 7,8 µl<br>E/RP Mix: 2,2 µl<br>Total: 10 µl	Mix: 15µl
**Reaction volume (total)**	20µl	25µl	20 µl	15 µl	20 µl
**Thermal cycling conditions**	RT: 50 °C (20 min); Activation: 95 °C (15 min); 45 cycles (95 °C 10 s / 60 °C 30 s); Extension: 72 °C (10 s)	RT: 50 °C (20 min); Activation: 95 °C (15 min); 45 cycles (95 °C 10 s / 60 °C 30 s); Extension: 72 °C (10 s)	UNG: 25 °C (2 min); RT: 50°C (10 min); Activation: 95 °C (2 min); 40 cycles (95 °C 30 s / 58 °C 30 s)	RT: 45 °C (15 min); Activation: 95 °C (2 min); 40 cycles (95 °C 15 s / 58 °C 30 s)	RT: 45 °C (15 min); Activation: 95 °C (3 min); 45 cycles (95 °C 15 s / 58 °C 60 s); Equipment: 25 °C (10 s)
**Reverse transcription format**	Two-Steps	One-Step	Two-Steps	One-Step	Two-Steps
**Fluorophores and quenchers**	FAN, ROX, VIC and Cy5	FAN, ROX, VIC and Cy5	RdRP	FAN and VIC	FAN, ROX and VIC
**Extraction Kits**	No RNA extraction	Quick-DNA/RNA™ Viral MagBead from Zymo Reaserch	No RNA extraction	Quick-DNA/RNA™ Viral MagBead from Zymo Reaserch	Quick-DNA/RNA™ Viral MagBead from Zymo Reaserch
